# Unraveling the role of disulfidptosis-related LncRNAs in colon cancer: a prognostic indicator for immunotherapy response, chemotherapy sensitivity, and insights into cell death mechanisms

**DOI:** 10.3389/fmolb.2023.1254232

**Published:** 2023-10-17

**Authors:** Hao Chi, Jinbang Huang, Yang Yan, Chenglu Jiang, Shengke Zhang, Haiqing Chen, Lai Jiang, Jieying Zhang, Qinghong Zhang, Guanhu Yang, Gang Tian

**Affiliations:** ^1^ Clinical Medical College, Southwest Medical University, Luzhou, China; ^2^ The Third Affiliated Hospital of Guizhou Medical University, Duyun, China; ^3^ First Teaching Hospital of Tianjin University of Traditional Chinese Medicine, Tianjin, China; ^4^ Heilongjiang University of Chinese Medicine, Harbin, Heilongjiang, China; ^5^ Department of Specialty Medicine, Ohio University, Athens, OH, United States; ^6^ Department of Laboratory Medicine, The Affiliated Hospital of Southwest Medical University, Luzhou, China

**Keywords:** Disulfidptosis, Colon cancer, LnRNAs, Tumor microenvironment, Prognostic markers, Immunotherapy response, Chemotherapy sensitivity

## Abstract

**Background:** Colon cancer, a prevalent and deadly malignancy worldwide, ranks as the third leading cause of cancer-related mortality. Disulfidptosis stress triggers a unique form of programmed cell death known as disulfidoptosis, characterized by excessive intracellular cystine accumulation. This study aimed to establish reliable bioindicators based on long non-coding RNAs (LncRNAs) associated with disulfidptosis-induced cell death, providing novel insights into immunotherapeutic response and prognostic assessment in patients with colon adenocarcinoma (COAD).

**Methods:** Univariate Cox proportional hazard analysis and Lasso regression analysis were performed to identify differentially expressed genes strongly associated with prognosis. Subsequently, a multifactorial model for prognostic risk assessment was developed using multiple Cox proportional hazard regression. Furthermore, we conducted comprehensive evaluations of the characteristics of disulfidptosis response-related LncRNAs, considering clinicopathological features, tumor microenvironment, and chemotherapy sensitivity. The expression levels of prognosis-related genes in COAD patients were validated using quantitative real-time fluorescence PCR (qRT-PCR). Additionally, the role of ZEB1-SA1 in colon cancer was investigated through CCK8 assays, wound healing experiment and transwell experiments.

**Results:** disulfidptosis response-related LncRNAs were identified as robust predictors of COAD prognosis. Multifactorial analysis revealed that the risk score derived from these LncRNAs served as an independent prognostic factor for COAD. Patients in the low-risk group exhibited superior overall survival (OS) compared to those in the high-risk group. Accordingly, our developed Nomogram prediction model, integrating clinical characteristics and risk scores, demonstrated excellent prognostic efficacy. *In vitro* experiments demonstrated that ZEB1-SA1 promoted the proliferation and migration of COAD cells.

**Conclusion:** Leveraging medical big data and artificial intelligence, we constructed a prediction model for disulfidptosis response-related LncRNAs based on the TCGA-COAD cohort, enabling accurate prognostic prediction in colon cancer patients. The implementation of this model in clinical practice can facilitate precise classification of COAD patients, identification of specific subgroups more likely to respond favorably to immunotherapy and chemotherapy, and inform the development of personalized treatment strategies for COAD patients based on scientific evidence.

## 1 Introduction

Colon cancer is a significant global health concern, which is defined as any malignant tumour originating from the inner epithelial layer of the colon (Rajamanickam and Agarwal, 2008), ranking as the third most prevalent cancer worldwide and leading to a substantial number of annual deaths (Wu et al., 2022). The incidence of Colon cancer is on the rise globally, with a particular increase observed in the Asian region. Projections by the World Health Organization indicate a further escalation in global Colon cancer cases, expected to reach 1.5 million by 2030 (Dmello et al., 2021). Due to the absence of early symptoms, most colon cancer patients are diagnosed in the intermediate or advanced stages, with little opportunity for radical surgery (Pan et al., 2021). With effective prevention in the past, colon cancer mortality rates have declined dramatically, but in 2008 it was estimated that there were 108,070 new cases of colon cancer and 49.960 colon cancer deaths (Rajamanickam and Agarwal, 2008). A survey of the prognosis of colon cancer patients shows that the 5-year survival rate for stage I colon cancer is 91 per cent, but drops to 72 per cent for locally advanced disease and to 14 per cent for stage IV colon cancer (Chetroiu et al., 2021). Cancer is a multifactorial, polygenic disease involving many complex biological processes in its development and progression. Presently, the primary modalities employed in the management of Colon cancer comprise surgical resection, chemotherapy, radiotherapy, and immunotherapy. Surgical intervention is considered the preferred treatment for patients with early-stage disease. Nevertheless, it should be noted that surgery may not offer curative outcomes for all individuals, particularly those diagnosed at advanced stages (Yao and Li, 2020). Recurrence and metastasis of Colon cancer make the treatment of this disease more challenging (Su et al., 2022). Currently, the TNM staging system is widely used for the prognostic assessment of Colon cancer patients. However, significant differences in patient survival time have been reported even at the same staging, suggesting that this assessment criterion is more limiting in predicting patient survival time (Yang et al., 2015). In view of this, it has become crucial to delve deeper into the pathogenesis of colon cancer, not only to better understand its origin and progression, but also to facilitate the emergence of more effective therapeutic strategies. A deeper understanding of the pathogenesis of malignant tumors holds the promise of innovative and improved therapeutic approaches. In addition, the search for novel colon cancer biomarkers is imperative for early detection and more effective treatment, which highlights the urgent need for new therapeutic pathways and predictive tools in the field of colon cancer treatment. Thus, this study explores the prognostic and therapeutic aspects of colon cancer based on the newly discovered biomarker.

Long non-coding RNAs (lncRNAs) are a class of RNA molecules characterized by their length of more than 200 nucleotides. Unlike messenger RNAs (mRNAs) that encode proteins, lncRNAs do not possess protein-coding capacity (Sun et al., 2019). Growing evidence suggests that dysregulated expression of lncRNAs is implicated in various diseases. Specifically, numerous studies have demonstrated that lncRNAs exhibit distinct expression patterns that are spatially, temporally, and cell-state specific, thereby playing pivotal roles in tumorigenesis and cancer progression (Yang et al., 2018). Growing evidence suggests that dysregulated expression of lncRNAs is implicated in various diseases. Specifically, numerous studies have demonstrated that lncRNAs exhibit distinct expression patterns that are spatially, temporally, and cell-state specific, thereby playing pivotal roles in tumorigenesis and cancer progression (Cai et al., 2022). Notably, emerging research has unveiled the significant involvement of lncRNAs in the pathogenesis of Colon cancer. Among the recently discovered programmed cell death pathways, disulfidoptosis has emerged as a distinctive mechanism triggering programmed cell death through specific signaling cascades (Liu X. et al., 2023). Therefore, studying disulfidptosisLncRNAs (DRLs) associated with disulfidptosis death may provide new predictive methods for tumor patient prognosis and immune microenvironment. Meanwhile, using techniques such as machine learning, accurate prediction models can be established to support clinical treatment and individualized medicine. However, the current research on disulfidptosis death-related lncRNAs in Colon cancer is still in the initial stage.

Cell death is a fundamental process that plays a crucial role in various biological activities, and its dysregulation is intimately linked to the development and progression of numerous diseases. The advantages and advances in bioinformatics and machine learning provide opportunities for a deeper and more comprehensive understanding of cell death and provide strong support for the development of new therapeutic approaches and personalized medicine. By applying a bioinformatics approach, we can identify and resolve genes and proteins associated with cell death, revealing their regulatory mechanisms and interaction networks. This systematic research approach can help us to comprehensively understand the molecular mechanisms of cell death and identify new therapeutic targets. Many important advances have been made in cell death research, driven by bioinformatics and machine learning. Medical big data and artificial intelligence have made significant progress and advantages in cancer research (Zhang P. et al., 2022; Zhao et al., 2022a; Zhao et al., 2022b; Zhao et al., 2022c; Zhang et al., 2023a). With the continuous development of data acquisition and storage technologies, more and more cancer dates are available for analysis and mining, which can help to discover patterns of disease progression and predict the risk of disease (Zhao et al., 2023a; Liu J. et al., 2023; Zhao et al., 2023b; Zhao et al., 2023c; Pei et al., 2023). Artificial intelligence is also playing an increasingly important role in cancer diagnosis, treatment, and prognosis, for example, in gene sequencing data analysis, cancer prediction, and personalized treatment (Cui et al., 2022; Zhao Z. J. et al., 2022). The advantages of medical big data and artificial intelligence are the ability to process large amounts of data, discover hidden patterns, and improve accuracy and personalized treatment, thus promising better medical care and treatment options for cancer patients (Zhang et al., 2023b). In this study, we have developed a predictive model for colon cancer Disulfidptosis Response-Related Long Non-Coding RNAs (DRLs) employing healthcare big data and artificial intelligence techniques. Leveraging the TCGA-COAD cohort, our model serves the purpose of predicting various facets of colon cancer prognosis, mutations, immune status, and responsiveness to chemotherapeutic drugs. Our methodology involved data mining of patient information from publicly available databases, followed by an extensive analysis encompassing enrichment, immunological, and drug sensitivity assessments. Additionally, we conducted internal validation and experimental verification, affirming the outstanding stability and reliability of the model we have constructed. This comprehensive approach offers a novel perspective for the implementation of precision and personalized treatment strategies in the management of tumors. The integration of medical big data and artificial intelligence provides a powerful framework to enhance our understanding of Colon cancer and guide tailored therapeutic interventions for improved patient outcomes. As technology continues to advance and research methods become more innovative, we believe the role of bioinformatics and machine learning in cell death research will be further enhanced, leading to greater breakthroughs in human health and disease treatment.

Our study represents a notable innovation and holds substantial scientific significance. Initially, we successfully identified a panel of long non-coding RNAs (lncRNAs) intricately linked to disulfidptosis-induced apoptosis in colon cancer (COAD) patients. Beyond RNA identification, our investigation advanced to encompass the development of a predictive model rooted in these lncRNAs. This model not only adeptly appraises COAD patient prognostics but also furnishes insights into their immune milieu. This pioneering approach is poised to furnish personalized therapeutic strategies tailored to COAD patients, with a particular emphasis on the realm of immunotherapy. Our model’s capacity to enhance clinicians’ comprehension of patients’ immune profiles promises more adept selection and optimization of treatment modalities, thereby augmenting the efficacy of immunotherapy. Of paramount significance, our findings illuminate the pivotal role played by lncRNAs in orchestrating immunotherapeutic responses, providing invaluable guidance for the formulation of more efficacious immunotherapy approaches. In sum, our study contributes noteworthy innovative and scientific value to COAD patient care and advances in tumor immunology.

## 2 Materials and methods

### 2.1 Data sources

Transcriptomic data and relevant clinical information of Colon adenocarcinoma (COAD) patients were retrieved from the TCGA database (https://portal.gdc.cancer.gov/). The data were accessed on 26 March 2023. Patients with missing samples in the Grade phase were excluded from the study. The remaining patients were randomly divided into training and test sets, maintaining a 7:3 ratio. Genes associated with disulfidoptosis were specifically selected for subsequent analysis. To distinguish between messenger RNA (mRNA) and long non-coding RNA (lncRNA), we utilized Strawberry Perl software, focusing on lncRNA-related data in this study. Clinical data including age, gender, tumor stage, and TNM stage were collected for the COAD patients.

### 2.2 Screening of DRLs

Differential gene expression analysis of COAD patients in TCGA was performed using the “limma” R package (Ritchie et al., 2015). Long non-coding RNAs (lncRNAs) with a standard deviation greater than 0.2 and a *t*-test *p*-value less than 0.05 were selected (Smyth, 2004). Subsequently, the correlation between the identified lncRNAs and disulfidoptosis data was calculated. LncRNAs with a correlation coefficient (corFilter) greater than or equal to 0.4 and a *p*-value (pvalueFilter) less than or equal to 0.001 were retained. The visualization of the results was achieved using the “dplyr” (Foster et al., 2018), “ggalluvial” (Brunson, 2020) and “ggplot2” (Ito et al., 2013) R packages, specifically utilizing the ggalluvial plots for enhanced visual representation.

### 2.3 Development and validation of prognostic models for DRLs

To integrate disulfidptosis response-related long non-coding RNAs (DRLs) and survival data in patients with Colon cancer (COAD), we employed the “limma” R package. Initially, we conducted univariate Cox regression analysis to identify differentially expressed DRLs significantly associated with prognosis. We set the significance threshold at a Cox *p*-value (coxPfilter) of less than 0.05. Based on these findings, we utilized the “caret” R package (Van E and ssen, 2012) to randomly divide the samples into training and test groups in a 7:3 ratio. Subsequently, we employed the “glmnet” R package (Engebretsen and Bohlin, 2019) to perform a minimum absolute shrinkage and selection operator (LASSO) Cox regression analysis, followed by a multivariate Cox regression analysis, to establish prognostic features. These features were then utilized to derive a risk score formula. The formula was calculated as follows: risk level = expressed lncRNA1 × CoeflncRNA1 + expressed lncRNA2 × CoeflncRNA2 + … + expressed lncRNAn × CoeflncRNAn, where Coefi represents the correlation coefficient. This study categorized COAD patients into two groups: a low-risk group and a high-risk group. To evaluate the prognostic efficacy of the derived risk score, we employed receiver operating characteristic (ROC) curves using multiple metrics. Internal validation was conducted by plotting ROC curves for both the training and test sets. Furthermore, we compared the overall survival of patients in the low-risk and high-risk groups using Kaplan-Meier survival curves. Additionally, we performed additional validation to assess the impact of clinical variables on prognostic prediction, including clinical ROC curves, C-index, and subgroup analysis.

### 2.4 Nomogram construction

To assess the ability of risk scores to serve as independent predictors and to develop a Nomogram for prognostic evaluation, we conducted univariate and multivariate Cox regression analyses. These analyses were performed using the TCGA-COAD cohort. To create a comprehensive visual representation, we utilized the “rms” package in R to construct a column line graph. This graph incorporated the risk scores along with clinicopathological characteristics to predict survival outcomes at specific time points, including 1, 3, and 5 years. The resulting Nomogram provides a useful tool for clinicians to estimate individual patient prognosis by considering the combined influence of risk scores and clinicopathological features.

### 2.5 Functional enrichment analysis

We conducted functional enrichment analysis for the differentially expressed genes associated with the four disulfidptosis response-related long non-coding RNAs (DRLs). This analysis aimed to annotate and investigate enriched pathways. To achieve this, we utilized ClusterProfiler, a widely used R package (Yu et al., 2012), to evaluate the KEGG pathway and gene ontology (GO) terms associated with the differentially expressed genes. By performing this analysis, we gained valuable insights into the functional roles and pathways associated with the identified genes. These findings provide a solid foundation for subsequent studies and further exploration of the molecular mechanisms underlying COAD.

### 2.6 Immunological analysis of risk characteristics

To evaluate the immune infiltration score associated with the risk characteristics, we employed multiple algorithms, including XCELL (Aran et al., 2017), TIMER (Li T. et al., 2020), QUANTISEQ (Chen et al., 2018), MCPCOUNT (Dienstmann et al., 2019), and EPIC (Racle et al., 2017). These algorithms allowed us to assess the immune cell composition within the tumor microenvironment. By utilizing these algorithms, we obtained comprehensive information on the infiltration levels of various immune cell types in relation to the identified risk characteristics. Furthermore, we compared the changes in immune checkpoints between patients in the high-risk and low-risk groups. Immune checkpoints play a crucial role in regulating the immune response, and alterations in their expression can influence tumor immune evasion. By examining the differences in immune checkpoint expression patterns, we gained insights into the potential immunological characteristics associated with the risk stratification.

### 2.7 Tumor mutation analysis

To obtain the tumor mutation load (TMB) data for COAD, we retrieved the information from the TCGA database. TMB represents the number of mutated bases per 1 million bases. The Strawberry Perl program was employed to collect and classify the TMB data into high and low categories, based on the TMB median. To further analyze the mutation profiles, we utilized the R package “maftool” (Mayakonda et al., 2018) to evaluate and present the 15 genes with the highest tumor mutation frequency (TMF) among COAD patients in the TCGA database. This analysis provided insights into the specific genes that exhibited a high frequency of mutations in COAD. Additionally, we compared the TMB levels between the high-risk and low-risk groups and performed log-rank tests for survival analysis. By examining the association between TMB and patient survival, we aimed to explore the potential impact of TMB on the prognosis of COAD patients.

### 2.8 Targeted drug sensitivity and immunotherapy response prediction

To predict the response to immunotherapy in COAD patients, we employed the Tumor Immune Dysfunction and Exclusion (TIDE) score. The TIDE score is a computational method that assesses the likelihood of response to immunotherapy based on the tumor’s immune microenvironment. By calculating the TIDE score for COAD patients, we aimed to predict their potential response to immunotherapy treatment. Furthermore, to predict the sensitivity of COAD patients to commonly used chemotherapeutic agents, we utilized the R package “oncoPredict” (Maeser et al., 2021). This package employs the half-maximal inhibitory concentration (IC50) values of COAD patients, which were obtained from the Genomics of Drug Sensitivity in Cancer (GDSC) database (Geeleher et al., 2014). By analyzing the IC50 data, we were able to predict the differential chemosensitivity of COAD patients to various chemotherapeutic agents commonly used in clinical practice.

### 2.9 Cell culture and qPCR assay

In our experimental study, we utilized several cell lines, including Caco-2 and SW480 colon cancer cells, along with NCM460 normal breast cells. These cell lines were sourced from the cell bank of the Central Laboratory of the Affiliated Hospital of Southwest Medical University. To ensure their proper growth and maintenance, we cultured these cells in DMEM (HyClone) medium supplemented with 10% fetal bovine serum (HyClone), 100 U/L penicillin, and 100 mg/L streptomycin (Thermo Fisher). We maintained standard culture conditions, which included a 5% CO2 atmosphere, to provide an optimal environment for cell viability and experimental consistency. For RNA extraction, we employed the RNA Eazy Fast Tissue/Cell Kit (TIANGEN Biotech Co.) following the manufacturer’s instructions. This kit facilitated the isolation of total RNA from the cell samples. Subsequently, the obtained RNA samples were utilized for cDNA synthesis using the FastKing RT Kit (TIANGEN Biotech Co.). Real-time PCR was performed on the StepOnePlus Real-Time PCR System (Thermo Fisher) using the SuperReal PreMix Plus reagent (TIANGEN Biotech Co.). The PCR protocol involved an initial pre-denaturation step at 95°C for 15 min, followed by 40 cycles of denaturation at 95°C for 10 s, annealing at 72°C for 20 s, and extension at 60°C for 20 s. For your reference, the primer sequences used in the PCR reaction can be found in Table 1.

**TABLE 1:** Gene primer sequences for 4 DRLs.

Gene	Sequences (5'–3')
H-ZEB1-AS1-F	GCT​GTT​GCT​TCT​CCC​TTC​TG
H-ZEB1-AS1-R	GTG​TCA​TCT​CCC​TTG​CCA​CT

### 2.10 Transient transfection

In our study, the colon cancer cell lines Caco-2 and SW480 were cultured using Dulbecco’s modified Eagle’s medium (DMEM; HyClone) supplemented with 10% fetal bovine serum (FBS; Hyclone), 100 U/L penicillin, and 100 mg/L streptomycin (Thermo Fisher). The cells were maintained at a temperature of 37°C in a 5% CO2 environment. For transient transfection experiments, we utilized Lipofectamine 3000 (Invitrogen, Carlsbad, CA, United States) as the transfection reagent. Negative Control (NC) and ZEB1-SA1 siRNA (RiboBio, Guangzhou, China) were transfected into the colon cancer cells according to the manufacturer’s instructions. This involved preparing the transfection mixture containing the siRNA and transfection reagent, which was then added to the cells. The transfection process was performed for a specified duration, typically following the manufacturer’s recommended protocol. By using Lipofectamine 3000 as the transfection reagent, we aimed to efficiently introduce the Negative Control or ZEB1-SA1 siRNA into the colon cancer cells, allowing for subsequent analysis and investigation of the effects of gene knockdown or control on cellular processes and molecular pathways.

### 2.11 CCK-8 assay

We assessed cell viability using the Cell Counting Kit-8 (CCK-8) assay. After 24 h of transfection, the colon cancer cells were seeded into 96-well plates at a density of 1,500 cells per well in 200 µL of complete medium. The cells were then incubated at 37°C. To perform the CCK-8 assay, 10 µL of CCK-8 reagent (Beyotime, Shanghai, China) was added to each well containing the cells. The plates were incubated for an additional 4 h at 37°C to allow the reagent to interact with the cells. During this time, the CCK-8 reagent undergoes a colorimetric reaction that correlates with cell viability. After the incubation period, the optical density value (OD450) was measured using a microplate reader. The OD450 measurement reflects the absorbance of the CCK-8 formazan product, which is directly proportional to the metabolic activity and viability of the cells. By quantifying the OD450 values, we were able to assess the relative cell viability and compare it across different experimental conditions or treatment groups.

### 2.12 Transwell assay

The invasion capabilities of colon cancer cells were evaluated using the Transwell assay, a well-established technique in cell biology research. In this assay, a specific number of colon cancer cells, approximately 1 × 10^5 cells, were seeded into specialized chambers. For assessing invasion potential, Matrigel-coated chambers were used. The upper chamber was filled with serum-free medium, creating a chemotactic gradient, while the lower chamber contained complete DMEM medium, providing a favorable environment for cell movement. Following an incubation period of 24 h, the cells that successfully invaded through the membrane were fixed using a 4% paraformaldehyde solution. To visualize and quantify the invaded cells, staining with 0.1% crystal violet was performed. The stained cells were then observed and counted under a light microscope, enabling the assessment of cell numbers and invasion capabilities.

### 2.13 Wound healing experiment

In order to evaluate the migratory capacity of colon cancer cells, a wound healing assay was employed. Cells post-transfection were cultivated in six-well plates and maintained at 37°C until they achieved an approximate confluency of 80%. Uniform wounds were introduced into the cell monolayer utilizing a 200 μL sterile pipette tip. After the creation of these wounds, the cells were rinsed twice with PBS to eliminate any remnants, and the medium was subsequently refreshed with a serum-free variant. Using an Olympus inverted microscope, the progression of cell migration to the injured area was recorded at 0 and 24 h. This allowed for a quantifiable assessment of the migration distance covered by the cells.

### 2.14 Statistical analysis

We employed R software version 4.2.3 and Strawberry Perl version 5.30.0 for statistical analyses. To assess the migration and invasion abilities of colon cancer cells, we conducted the Transwell assay. Cells were seeded into Matrigel-coated or uncoated chambers for invasion or migration, respectively. Following 24 h of incubation, cells that migrated or invaded through the membrane were fixed and stained. Quantification was performed using a light microscope. In addition, we used Kaplan-Meier survival curves and log-rank tests to compare the overall survival of high-risk and low-risk groups. Prognostic characteristics were constructed using the LASSO Cox regression model, and their predictive power was evaluated using ROC curves. Differences in tumor-infiltrating immune cells, immune checkpoints, and immune function were assessed using the Wilcoxon test. Significance thresholds were set at *p* < 0.05 and FDR <0.05 to ensure robust statistical analysis with low false discovery rates.

## 3 Results

### 3.1 Identification of candidate disulfidptosis death-associated LncRNAs

**FIGURE 1:**
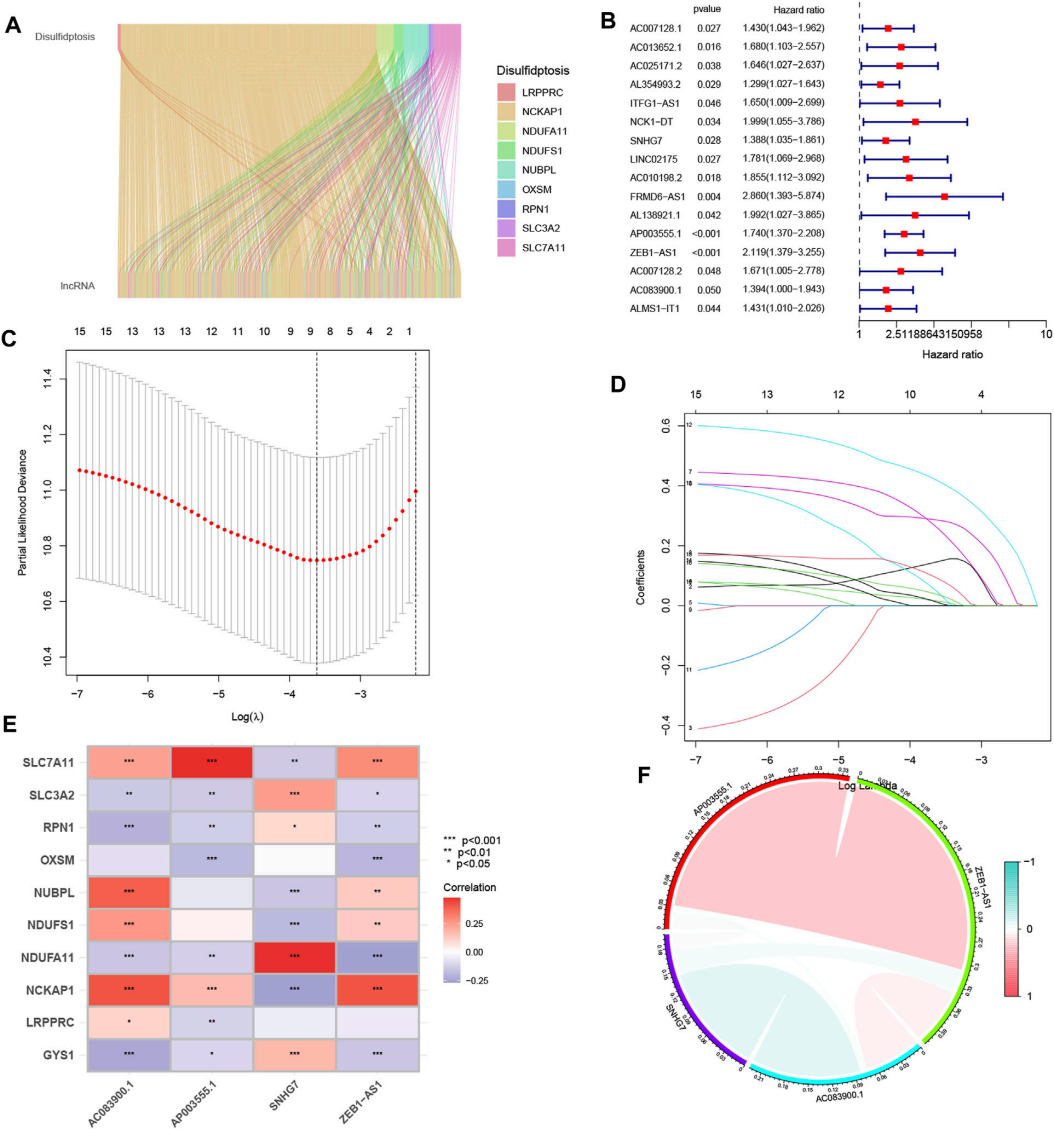
The identification process of candidate long non-coding RNAs (lncRNAs) associated with disulfidptosis. **(A)** The ggalluvial plot displays the co-expression of 802 lncRNAs and their associated genes related to disulfidptosis. **(B)** Univariate Cox regression analysis is employed to evaluate the prognostic significance of the disulfidptosis-associated lncRNAs. **(C)** The Lasso model is adjusted for parameter selection using tenfold cross-validation. **(D)** Lasso coefficient curves are depicted. **(E)** The heat map visualizes the correlation between the four identified lncRNAs and the disulfidptosis-associated genes. **(F)** A correlation study examines the relationship among the four lncRNAs associated with disulfidptosis. *p < 0.05; **p < 0.01; ***p < 0.001.

The analysis identified ten genes associated with disulfidptosis (Liu X. et al., 2023). Co-expression analysis was performed between these genes and disulfidptosis-associated lncRNAs, resulting in the identification of 802 lncRNAs showing co-expression patterns (Figure 1A). Further one-way Cox analysis led to the identification of high-risk lncRNAs associated with disulfidptosis (Figure 1B). Subsequently, the Lasso algorithm was applied to these lncRNAs, revealing nine lncRNAs with the least cross-validation error (Figures 1C, D). Among them, four relevant DRLs, namely, AC083900.1, AP003555.1, SNHG7, and ZEB1-AS1, were identified based on their regression coefficients (0.4138, 0.4786, 0.4745, and 0.4086, respectively). A multifactorial Cox proportional risk regression model was employed to scale down the high-dimensional data, and a risk score formula was derived as follows: risk score = (0.4138 × AC083900.1 expression level) + (0.4786 × AP003555.1 expression level) + (0.4745 × SNHG7 expression level) + (0.4086 × ZEB1-AS1 expression level). Further analysis demonstrated strong correlations between the genes associated with disulfidptosis and the identified DRLs (Figure 1E), as well as strong correlations among the four DRLs themselves (Figure 1F).

### 3.2 Model construction and testing of disease predictive value

**FIGURE 2:**
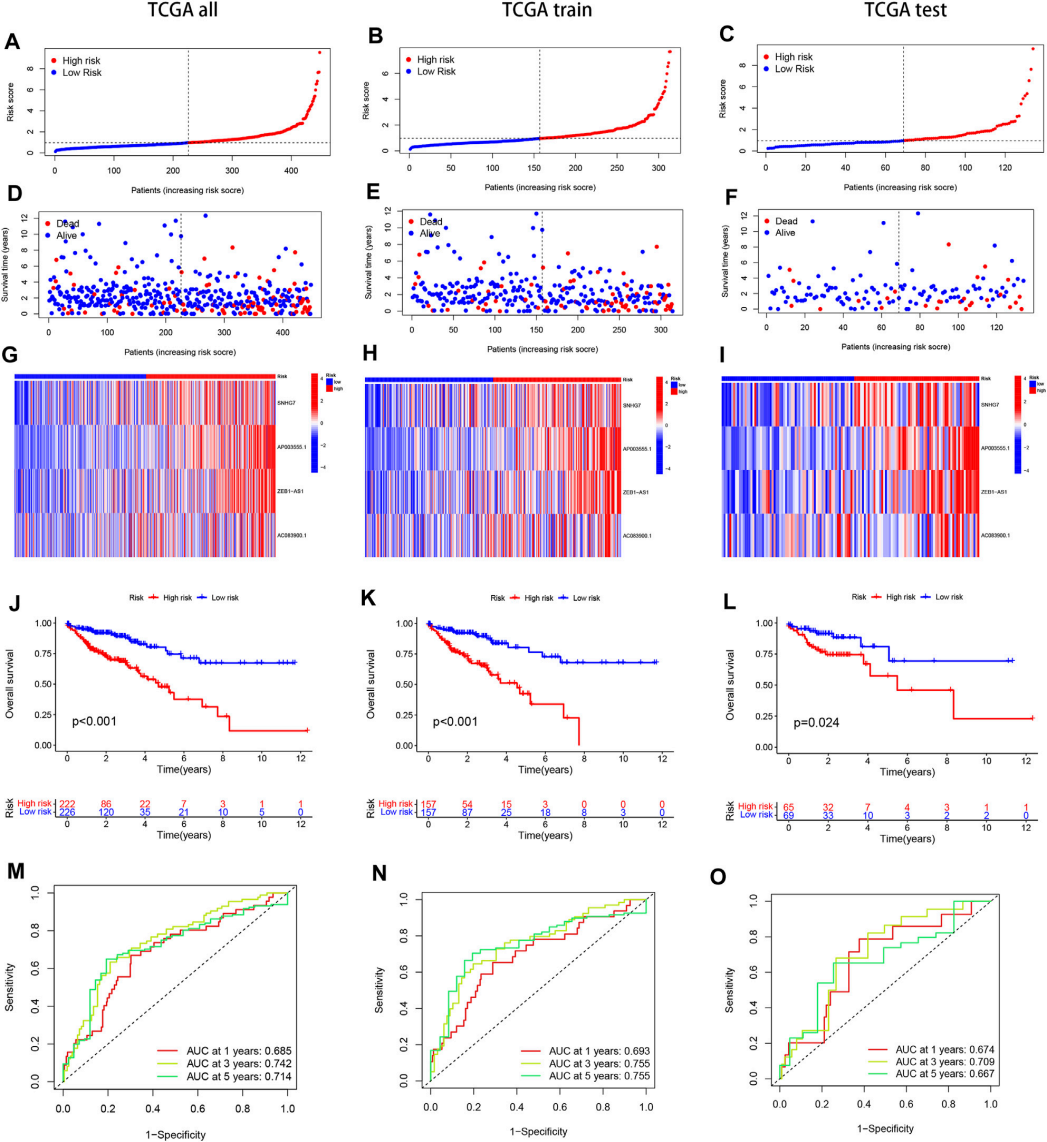
Present the Kaplan-Meier (KM) curves of risk models in various datasets, including the training and test groups. **(A–C)** The risk scores distribution is shown for patients with COAD. **(D–F)** The survival time and status distribution of COAD patients in the low- and high-risk populations is displayed. **(G–I)** A heat map illustrates the expressions of the four identified lncRNAs. **(J–L)** The overall survival (OS) curves are presented for high-risk and low-risk patients in different groups. **(M–O)** The area under the curve (AUC) is depicted for 1-year, 3-year, and 5-year survival times in different groups to assess the sensitivity and specificity of the prognostic model.

We divided the samples into a Train group and a Test group in a 7:3 ratio to construct the prognostic model and verify its accuracy. The risk score for each sample was calculated by multiplying the expression of the four selected disulfidptosis-related lncRNAs by their corresponding regression coefficients. The samples were then sorted based on the risk scores, and the high and low-risk groups were defined using the median score. Survival analysis of COAD patients in the TCGA cohort showed that elevated risk scores were associated with poorer survival outcomes (Figures 2A–F). A heat map was generated to visualize the expression patterns of the four lncRNAs in the high and low-risk groups across the three datasets (whole, test, and training), revealing distinct expression patterns (Figures 2G–I). These findings support the identification of the four lncRNAs as risk factors. Kaplan-Meier analysis demonstrated significant differences in survival between the high-risk and low-risk groups across the entire dataset, test subset, and training subset (Figures 2J–L). The constructed model showed good predictive accuracy, as indicated by the area under the curve (AUC) values of 0.685, 0.742, and 0.714 for 1-year, 3-year, and 5-year survival, respectively, in the entire COAD dataset (Figure 2M). Internal validation using randomly grouped training and test sets further confirmed the accuracy of the model, with AUC values of 0.693, 0.755, and 0.755 for the training group and 0.674, 0.709, and 0.667 for the validation group at 1, 3, and 5 years, respectively (Figures 2M–O). These results demonstrate the high accuracy and sensitivity of the prognostic model for predicting survival outcomes in COAD patients.

### 3.3 Principal component analysis

**FIGURE 3:**
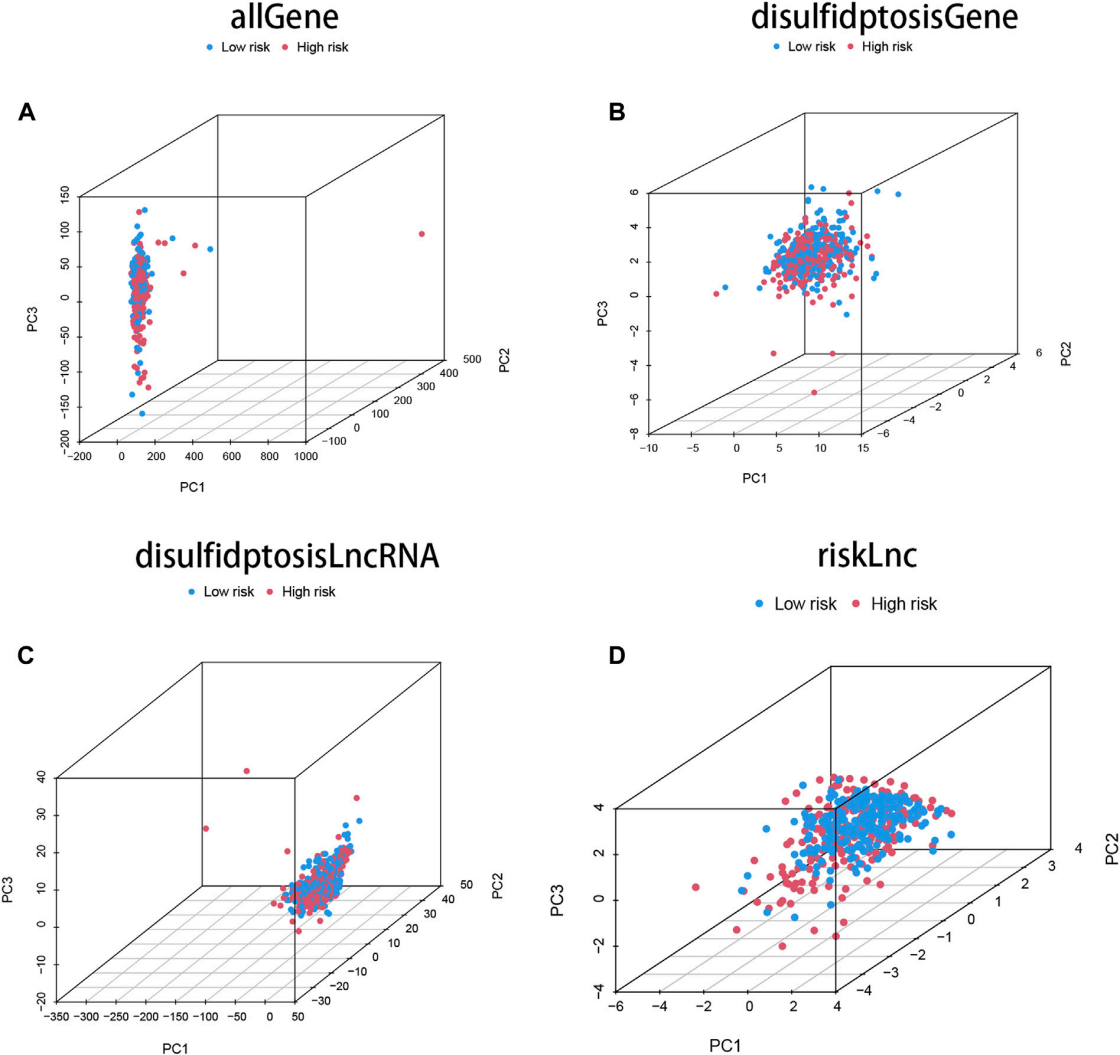
The characteristics of DRLs enable better differentiation between high- and low-risk groups of patients. **(A)** Principal component analysis (PCA) profiles of all genes demonstrate the overall gene expression patterns. **(B)** PCA profiles of disulfidptosis-associated genes specifically highlight the expression patterns of genes associated with disulfidptosis. **(C)** PCA profiles of DRLs specifically focus on the expression patterns of the identified long non-coding RNAs. **(D)** The PCA profiles of lncRNAs were generated using riskLnc features for further analysis.

Principal component analysis (PCA) was performed on four different gene sets, including all genes, disulfidptosis-associated genes, disulfidptosis-associated lncRNAs, and model lncRNAs, to evaluate their respective contributions to the risk model. The PCA plots for these gene sets are shown in Figures 3A–D. Notably, the PCA plot of the risk lncRNAs revealed distinct separation between high and low-risk patients, indicating significant differences and successful stratification into two relatively independent clusters (Figure 3D). These findings provide further validation for the effectiveness of our method in accurately distinguishing between low and high-risk populations. Moreover, they demonstrate the robustness, generalizability, and predictive power of the risk model in COAD patients.

### 3.4 Correlation analysis between DRLs and clinicopathological characteristics

**FIGURE 4:**
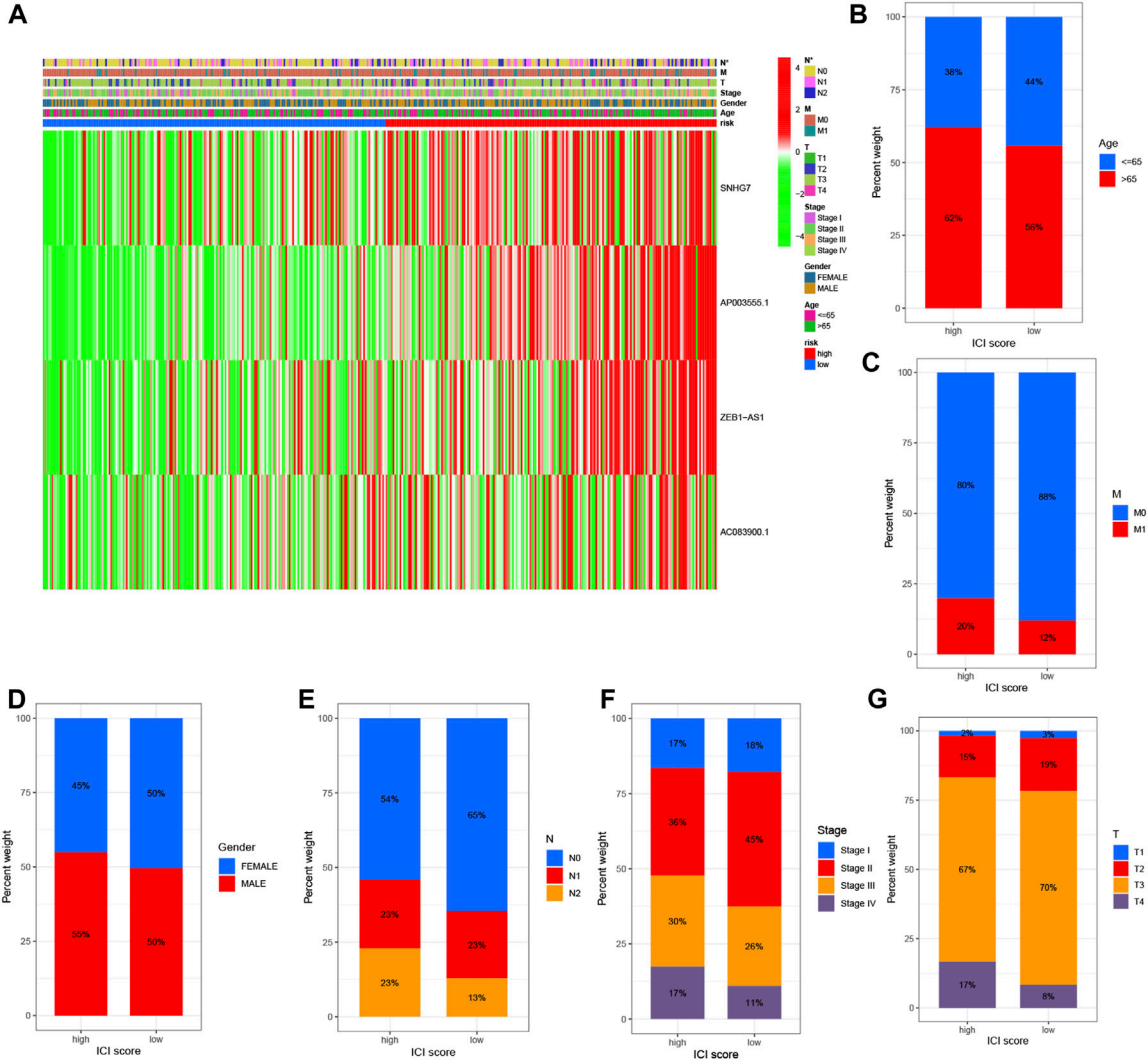
Correlation analysis of DRLs with clinicopathological characteristics reveals their association with disease-related factors. **(A)** The heat map illustrates the relationship between risk scores based on DRLs and various clinicopathological characteristics. Bar graphs depict the differences in patient distribution between the high-risk and low-risk groups stratified by different pathological characteristics, including **(B)** age, **(C)** M-stage, **(D)** gender, **(E)** N-stage, **(F)** stage, and **(G)** T-stage.

To examine the association between high and low-risk groups and various clinical features, we generated a heat map displaying the correlation between these groups and clinical characteristics such as age, gender, stage, T stage, N stage, M stage, and risk scores (Figure 4A). This comprehensive analysis incorporated data from all TCGA Colon cell carcinoma patient samples. Furthermore, we investigated the proportional differences in different clinical traits, including age, gender, survival status, T stage, M stage, and N stage, between the high and low-risk groups. Interestingly, we observed distinct variations in the distribution of patients with different clinicopathological traits between the high and low-risk groups. Notably, the four identified DRLs exerted a significant influence on the frequency of specific clinicopathological traits, highlighting their potential clinical relevance (Figures 4B–G). These findings provide valuable insights into the relationship between the risk model and clinical characteristics, shedding light on potential associations and implications in COAD patients.

### 3.5 Clinical subgroup analysis of disulfidptosis-related lncRNA model

**FIGURE 5:**
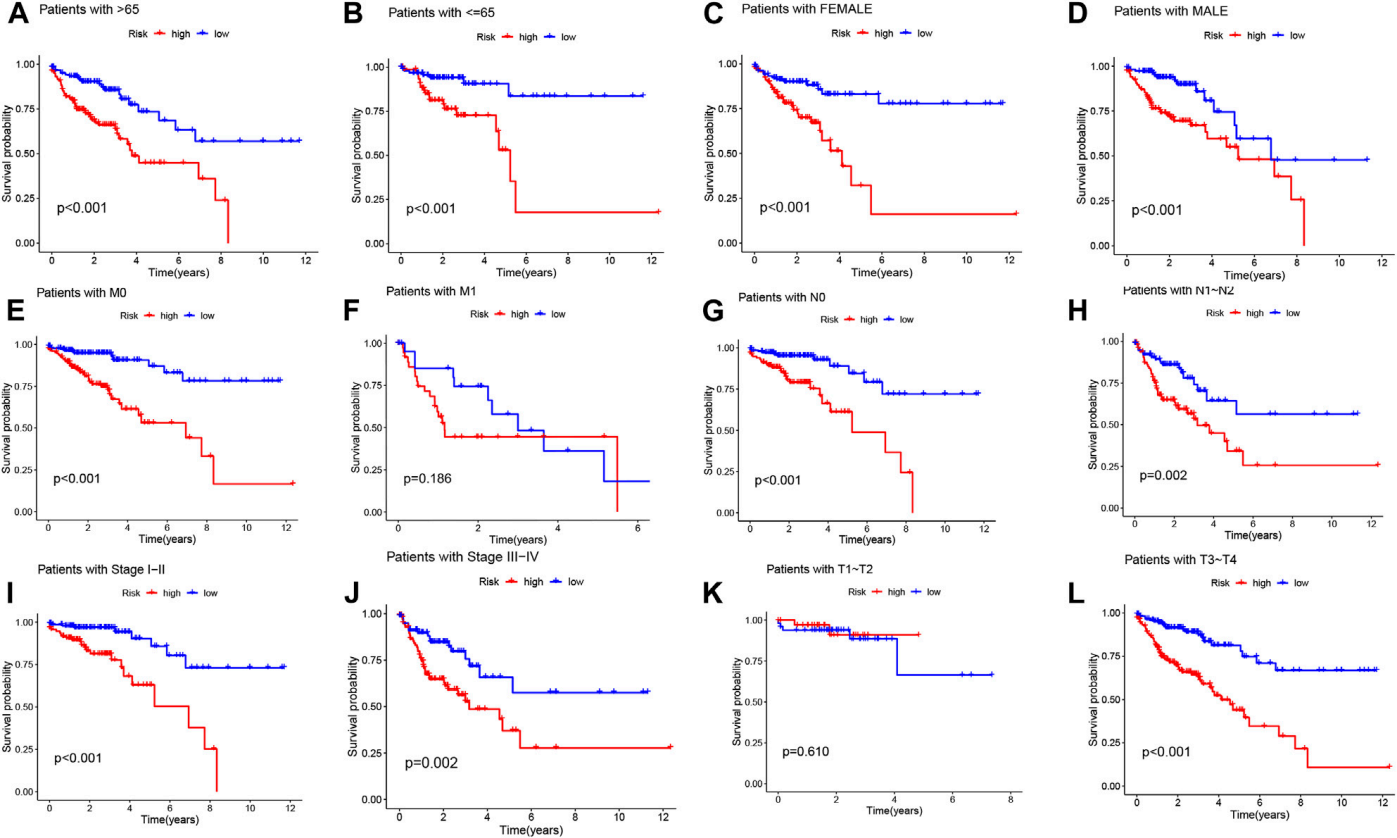
Kaplan-Meier survival curves demonstrate the prognostic value of the risk model in COAD patients, stratified by various clinical characteristics. **(A–L)** The figures present Kaplan-Meier curves for patients with COAD in the low- and high-risk groups, categorized based on different clinical characteristics.

To further investigate the impact of the risk model on patient prognosis within specific clinical subgroups, we divided COAD patients into six distinct subgroups based on age (>65 and ≤65 years), gender (male and female), M-stage (M0 and M1), N-stage (N0 and N1-2), clinical stage (I-II and III-IV), and T-stage (T1-T2 and T3-T4). Survival curves were then analyzed and compared between the high- and low-risk groups within each subgroup (Figures 5A–L). Our findings revealed that, except for patients with M1 and T1-T2 stage, the overall survival rate of high-risk patients was significantly lower than that of low-risk patients across all subgroups. These results indicate that low-risk patients exhibit a distinct survival advantage. Collectively, our analysis demonstrates the robustness and reliability of the DRLs risk model as a valuable clinical prediction tool, capable of accurately predicting the prognosis of COAD patients across different clinical subgroups.

### 3.6 Independent prognostic analysis of clinical characteristics and Nomogram construction

**FIGURE 6:**
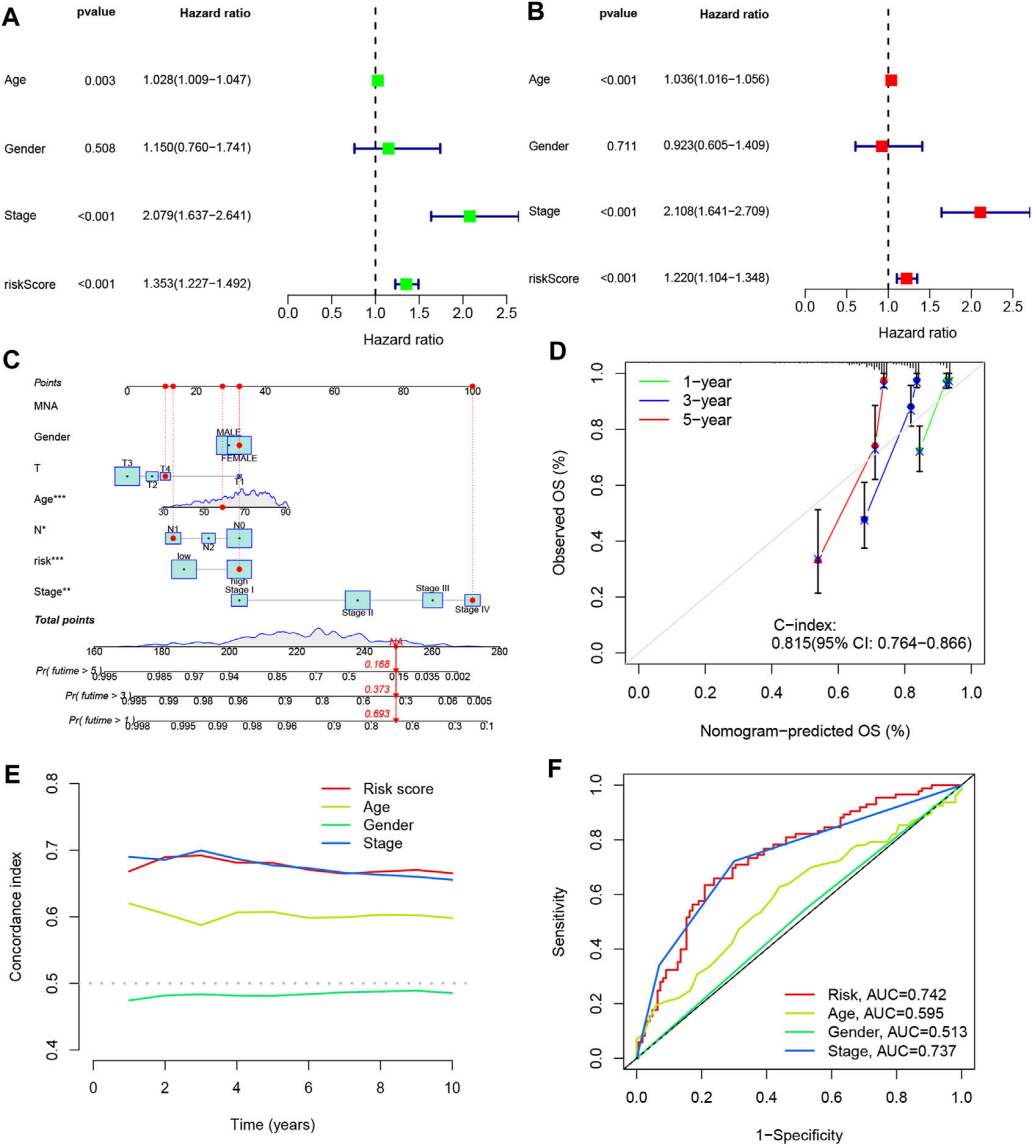
Line plots were created to analyze the independent prognostic factors in COAD patients. **(A, B)** Single Cox and multiple Cox regression analyses were performed to identify significant factors. **(C)** Column line graphs were used to assess overall survival (OS) in COAD patients at one, three, and 5 years. **(D)** Calibration curves were generated to evaluate the performance of the column line graphs. **(E)** C-index curves were plotted to assess the predictive accuracy of different characteristics. **(F)** ROC curves were constructed to evaluate the discriminatory power of various characteristics.

To assess the prognostic value of the four DRLs in relation to common clinical characteristics, we conducted both univariate and multifactorial Cox analyses. In the univariate analysis, age (*p* = 0.003), stage (*p* < 0.001), and risk score (*p* < 0.001) were found to be significantly associated with the prognosis of COAD patients (Figure 6A). Subsequently, a multifactorial Cox analysis was performed, which revealed that age (*p* < 0.001), staging (*p* < 0.001), and risk score (*p* < 0.001) remained as reliable and independent predictors of risk (Figure 6B). To enhance the clinical applicability of the risk model, we constructed a column line plot incorporating sex, age, staging, and risk score to predict the probability of prognostic survival at 1, 3, and 5 years (Figure 6C). Notably, the risk score exhibited the most significant impact on the prediction of overall survival (OS), suggesting that COAD prognosis can be more accurately predicted using this risk model. Furthermore, calibration curves demonstrated favorable agreement between predicted and observed values for the probability of OS at 1, 3, and 5 years, indicating the stability of the Nomogram plot (Figure 6D). The c-index values and area under the ROC curve for the risk score surpassed those of other clinical traits, underscoring the superior accuracy of the constructed model in predicting patient survival (Figures 6E, F).

### 3.7 Enrichment analysis of COAD patients based on prognostic markers

**FIGURE 7:**
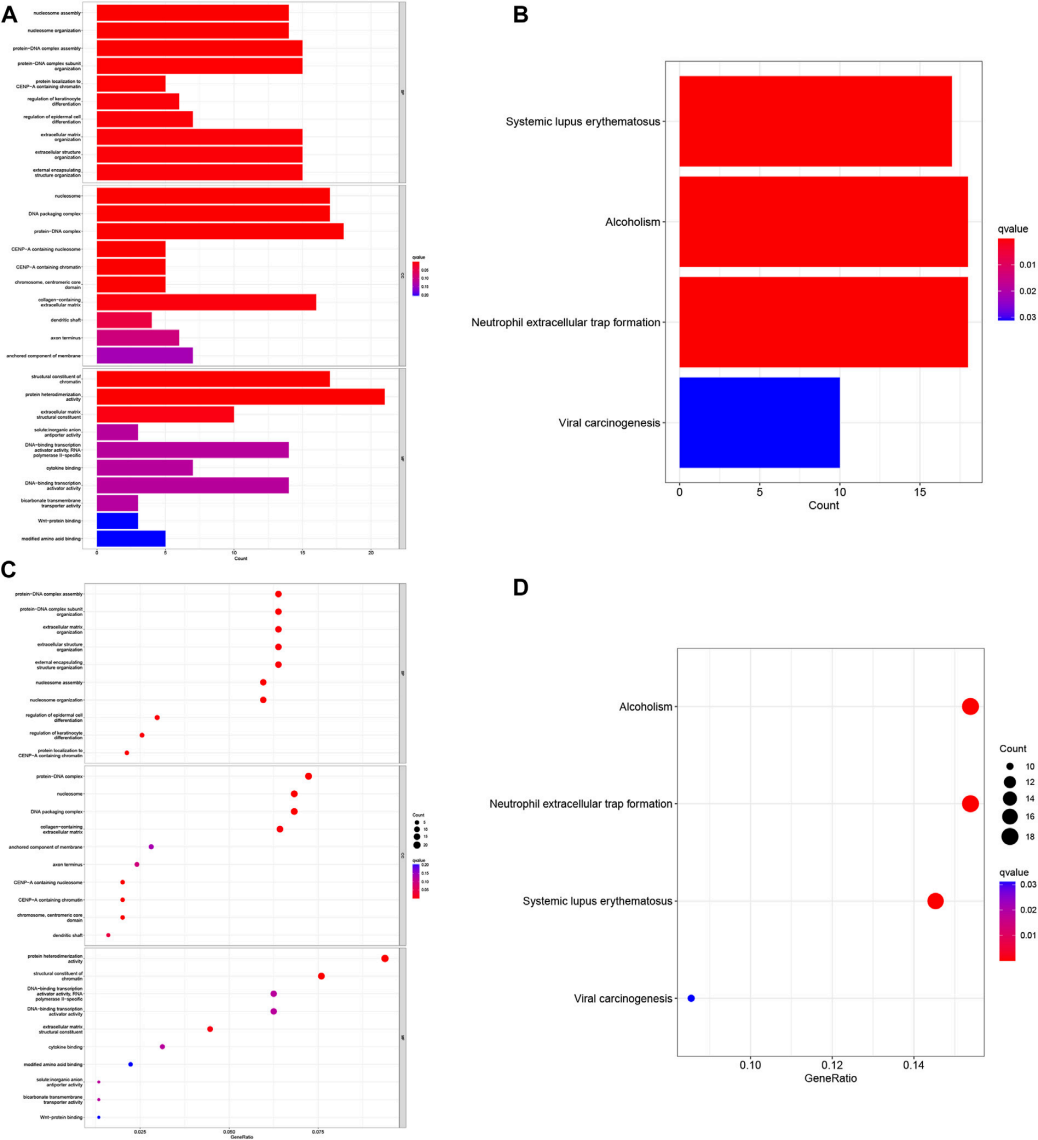
Gene Ontology (GO) and Kyoto Encyclopedia of Genes and Genomes (KEGG) pathway enrichment analysis. **(A)** Histogram of the 10 most enriched words of GO. **(B)** Histogram of the top 4 most enriched words of KEGG. **(C)** Bubble chart of the top 10 GO-rich 10 words. **(D)** Bubble chart of the 4 richest words in KEGG.

To investigate the functional relevance and signaling pathways associated with risk scores, we conducted GO functional analysis and KEGG enrichment analysis on differentially expressed genes between the high- and low-risk groups. Significantly enriched terms were identified based on the thresholds of FDR<0.05 and *p* < 0.05. Notably, we observed distinct enrichment results between the high- and low-risk groups. GO enrichment analysis revealed that in terms of biological processes (BP), the high-risk group exhibited significant enrichment in protein-DNA complex assembly, protein-DNA complex subunit organization, extracellular matrix organization, and external encapsulated structure organization. Cellular components (CC) were predominantly enriched in protein-DNA complexes, DNA packaging complexes, nucleosomes, and related structures. Molecular functions (MF) were mainly associated with the structural components of protein heterodimer activity and pigments (Figures 7A–C). In addition, the KEGG pathway analysis revealed significant enrichment in pathways related to systemic lupus erythematosus (SLE), alcoholism, and neutrophil extracellular traps in cancer (Figures 7B–D). These findings highlight differences in immune function-related pathways between the low- and high-risk categories. Subsequently, we performed immunological studies on the two subgroups of COAD, further exploring their implications in immune function and potential therapeutic targets.

### 3.8 Predictive analysis of the correlation between immune cell infiltration and tumor microenvironment by disulfidptosis-associated lncRNA model

**FIGURE 8:**
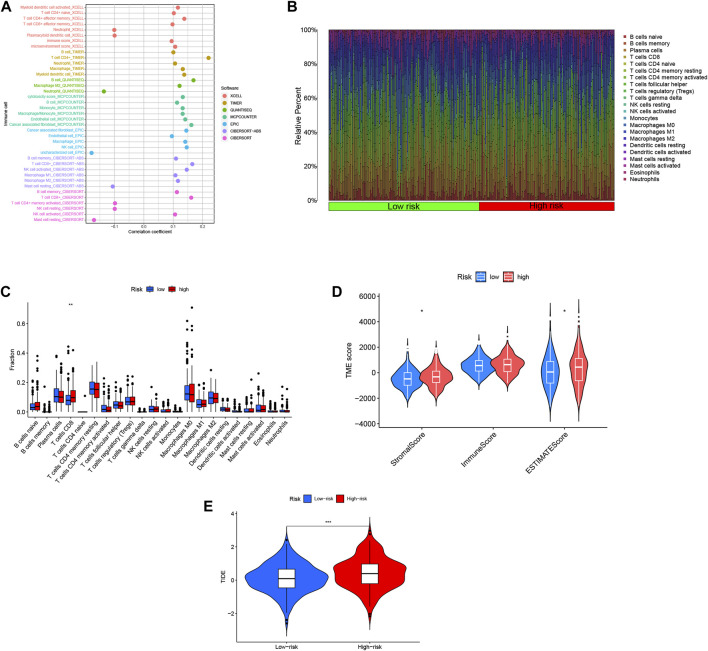
Risk scores for DRLs were utilized to predict the tumor microenvironment and immunotherapy outcomes. **(A)** An immune cell bubble plot was generated. **(B)** A histogram depicted the proportion of 22 tumor-infiltrating immune cell types in COAD tumor samples. **(C)** Differences in different types of immune cells between high- and low-risk groups. **(D)** Differences in tumor microenvironment (TME) scores between high- and low-risk groups were examined. **(E)** Tumor Immune Dysfunction and Exclusion (TIDE) Variations in Tumor Immune Dysfunction and Exclusion (TIDE) scores between the high-risk and low-risk groups were evaluated.

In order to investigate the relationship between the risk score and the tumor microenvironment (TME), we assessed the abundance of tumor-infiltrating immune cells (TIIC) using various algorithms including XCELL, TIMER, QUANTISEQ, MCPCOUNTER EPIC, CIBERSORT-ABS, and CIBERSORT (Fan et al., 2021). We observed a positive correlation between the risk score and the infiltration of most immune cell types, particularly CD4^+^ T cells and CD8^+^ T cells (Figure 8A). This suggests that higher risk scores are associated with increased immune cell infiltration in COAD. To gain a comprehensive understanding of the immune landscape in COAD, we utilized the CIBERSORT algorithm to explore the infiltration of 22 immune cell subpopulations based on mRNA expression in TCGA COAD patient tissues. The bar graph representation showed varying levels of infiltration for different immune cell populations, with relatively high percentages of M0, M1, and M2 macrophages, as well as CD8^+^ T cells and CD4^+^ T cells (Figure 8B). Further analysis of the differences between the high- and low-risk groups revealed that CD8^+^ T cells were the only immune cell population that exhibited a statistically significant difference, with higher levels in the high-risk group compared to the low-risk group (Figure 8C). This suggests a potential association between high-risk scores and increased infiltration of CD8^+^ T cells. Moreover, we evaluated the stromal cell scoring and stromal cell immune cell integrated scoring in the tumor microenvironment between the high- and low-risk groups. The results showed that the stromal cell scoring and stromal cell immune cell integrated scoring were higher in the high-risk group, indicating a higher abundance of stromal cells in this group (Figure 8D). Additionally, we employed the Tumor Immunosuppression and Rejection (TIDE) algorithm to estimate the likelihood of response to immunotherapy based on the risk model. The TIDE scores were significantly higher in the high-risk group compared to the low-risk group (*p* < 0.05), suggesting that high-risk patients may have a higher chance of evading immunization, potentially leading to a poorer response to immunotherapy (Figure 8E). These findings provide insights into the immune cell composition and tumor microenvironment characteristics associated with the risk scores in COAD, highlighting potential implications for immunotherapy and understanding the immune phenotype of the disease.

### 3.9 Differential analysis of lncRNAs drug sensitivity associated with disulfidptosis

**FIGURE 9:**
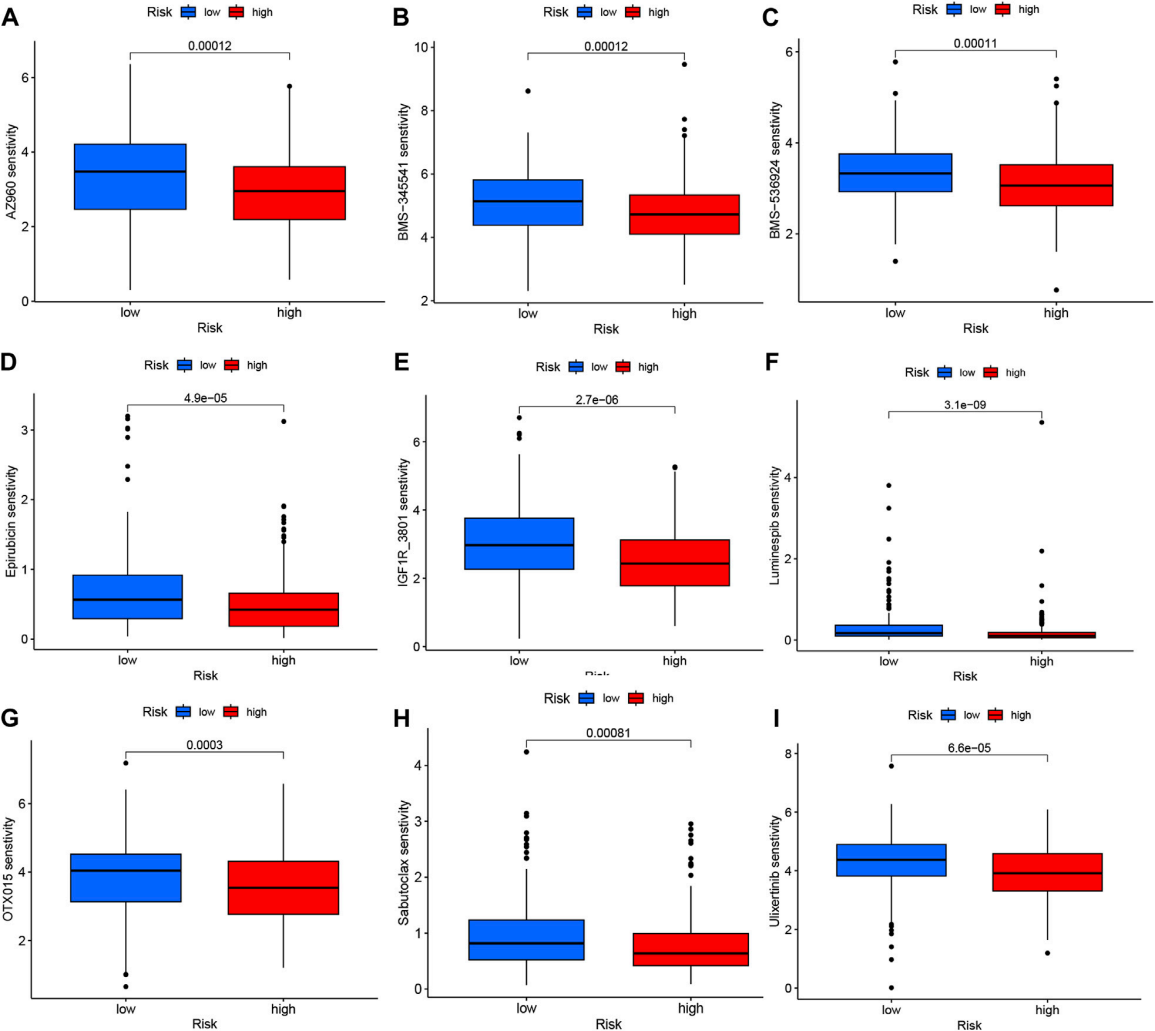
Variations in IC50 values of different immunotherapy drugs were examined based on risk scores: **(A)** AZ960, **(B)** BMS-345541, **(C)** BMS-536924, **(D)** Epirubicin, **(E)** IGF1R_3801, **(F)** Luminespib, **(G)** OTX015, **(H)** Sabutoclax, and **(I)** Ulixertinib.

The analysis of immunotherapeutic agents in relation to the risk score provided insights into the potential efficacy of immunotherapy for COAD patients and the potential for dose adjustment. Among the nine immunotherapeutic agents evaluated, all of them showed significant differences (*p* < 0.05) between the high- and low-risk groups. Specifically, the low-risk group exhibited higher IC50 values, indicating lower sensitivity to the immunotherapeutic agents, while the high-risk group demonstrated greater sensitivity to these drugs (Figures 9A–I). This information suggests that COAD patients with higher risk scores may have a more favorable response to immunotherapy, as indicated by their increased sensitivity to the immunotherapeutic agents tested. The risk score can be utilized to further investigate and enhance precise drug therapy for immunotherapy in COAD patients. This analysis highlights the potential utility of the risk model in guiding treatment decisions and optimizing immunotherapeutic approaches.

### 3.10 Comparison of somatic mutations in high and low-risk groups

**FIGURE 10:**
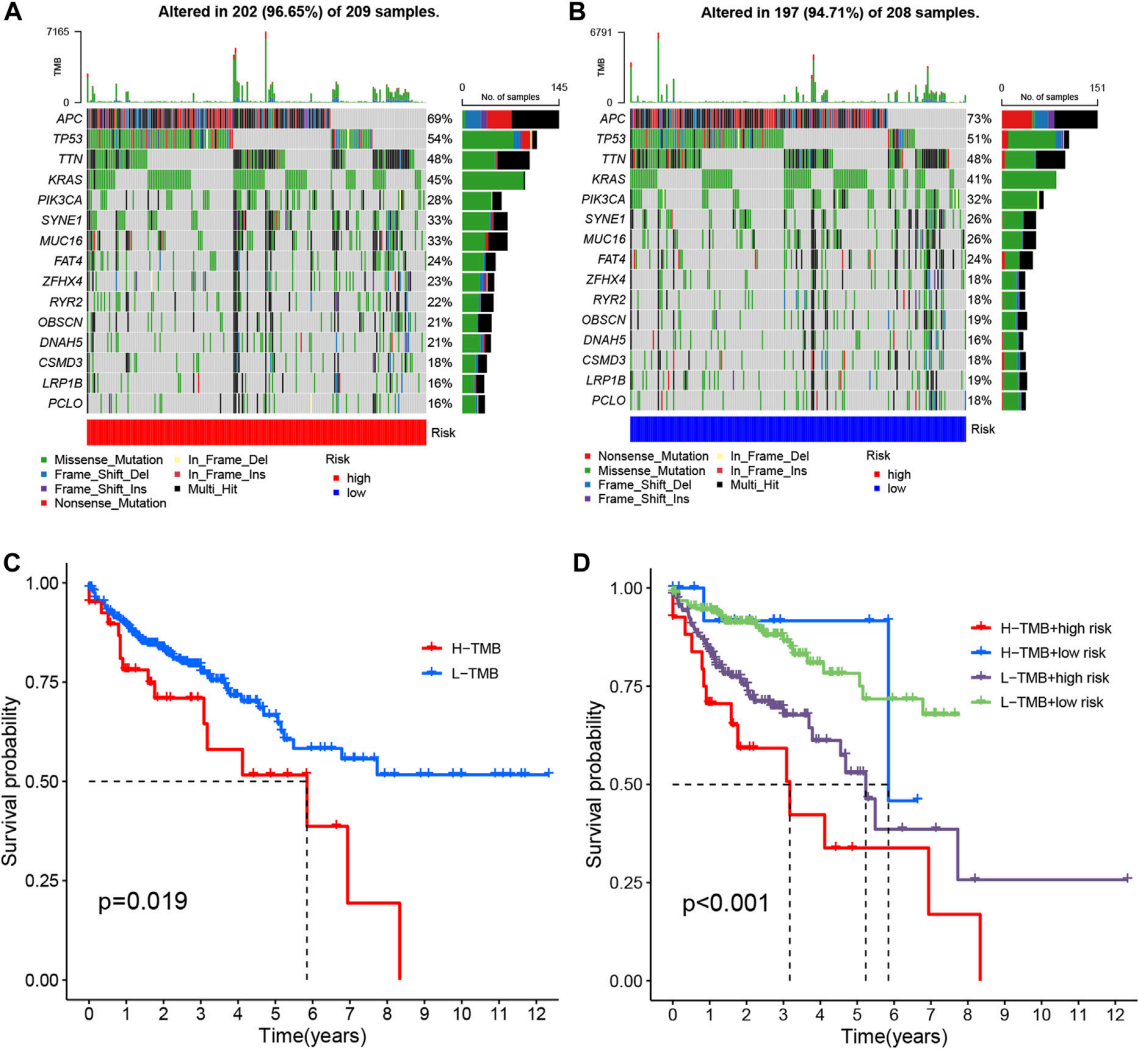
The mutation analysis of COAD tumors. **(A, B)** The top 15 most prevalent mutated genes in high-risk and low-risk patients are depicted. **(C)** The overall survival curves demonstrate the differences between the high mutation group and low mutation group. **(D)** The overall survival curves compare the four different subgroups.

Tumor mutational burden (TMB) quantifies the number of non-synonymous mutations occurring within specific genomic regions of somatic cells. It serves as an indirect indicator of the tumor’s potential to generate neoantigens, reflecting its immunotherapy responsiveness (Huang et al., 2023). To investigate TMB in COAD patients, we conducted an analysis on somatic mutation data, classifying individuals into high-risk and low-risk groups (Figures 10A, B). Notably, approximately 96.65% of COAD patients in the high-risk group exhibited mutations, with APC (69%), TP53 (54%), and TTN (48%) being the most frequently mutated genes. Similarly, in the low-risk group, 94.71% of COAD patients demonstrated mutations, wherein APC (73%), TP53 (51%), and TTN (48%) were the top three genes affected.

The APC genes play a pivotal role as oncogenes by governing cellular growth and maintaining stability. In tumor tissues of individuals with colon cancer, APC genes are frequently subjected to high levels of methylation. This methylation phenomenon exerts a significant influence on the proliferation and invasive potential of colon cancer cells, and is also associated with clinical parameters including tumor size and degree of differentiation (Li et al., 2017). Excitingly, the administration of demethylating agents such as 5-aza-CdR (Sanaei et al., 2020), procaine, and carboplatin (Sabit et al., 2016) has shown promising therapeutic efficacy in patients with colon cancer.

To compare the tumor mutational loads between the two groups, we analyzed the somatic mutation data and determined the TMB for each group. Notably, the low-risk group exhibited a lower TMB compared to the high-risk group (Figure 10C) (Lakens, 2015). Subsequently, we performed a survival analysis by categorizing patients into two groups based on the median TMB of each sample and employing the Kaplan-Meier test. The results revealed a significant difference in patient survival between the high and low mutational load groups, with a more favorable prognosis observed in the low mutational load group (*p* = 0.019) (Figure 10D). This finding may be attributed to the potential of high TMB to trigger anti-tumor immune responses. Furthermore, when we combined the tumor’s mutational load with the risk level of the sample and performed a survival analysis, we observed that patients with both high-risk and high TMB had the lowest overall survival (*p* < 0.001) (Figure 10E). Consequently, our risk model, in conjunction with TMB, holds promise for more accurate prognostic predictions in COAD patients.

### 3.11 ZEB1-SA1 promotes the proliferation and migration of colon cancer cells

**FIGURE 11:**
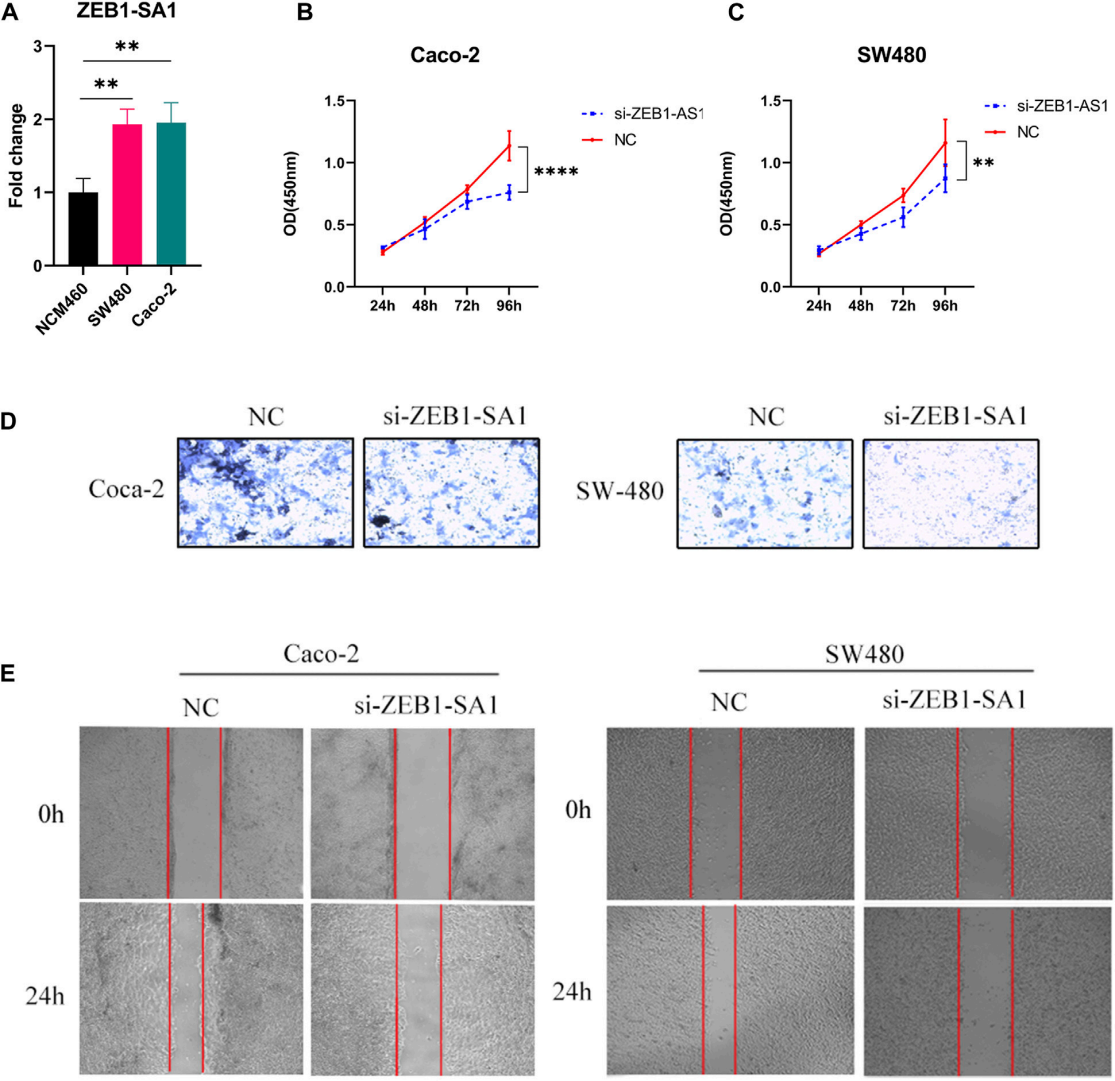
ZEB1-AS1 has been shown to enhance the proliferation, invasion, and migration abilities of colon cancer cells. This was evaluated through various assays: **(A)** qPCR assay, **(B, C)** CCK-8 assay, **(D)** Transwell assay, and **(E)** wound healing experiment.

Validated by qPCR, we observed elevated expression levels of ZEB1-SA1 in colon cancer cells, which was consistent with the results of bioinformatics analysis (Figure 11A). To investigate the potential role of ZEB1-SA1 in colon cancer, we conducted *in vitro* experiments. Firstly, the CCK-8 assay demonstrated that silencing ZEB1-SA1 led to a notable inhibition of cell proliferation (Figures 11B, C). Additionally, transwell assays and wound healing experiment revealed that knockdown of ZEB1-SA1 significantly attenuated the invasive and migratory abilities of the cells (Figures 11D, E). Collectively, these results suggest that ZEB1-SA1 functions as an oncogene, and its upregulation promotes the proliferation, invasion, and migration of colon cancer cells.

## 4 Discussion

Colon cancer is a prevalent and deadly malignancy arising from endothelial cells in the Colon. Its global incidence has been steadily increasing, with approximately 1.4 million new cases and nearly 700,000 deaths reported annually (Li et al., 2022). Metastasis, observed in 20%–25% of colon cancer patients at diagnosis, is associated with poorer prognosis and increased treatment challenges, often necessitating a combination of radiotherapy and chemotherapy (Chang et al., 2021). However, other treatment options are not as effective as chemotherapeutic drugs as currently known. Once patients become resistant to chemotherapy regimens, it will pose a significant challenge to treatment (Li et al., 2011). After research, it has been found that biomarkers of Colon cancer can help physicians to develop a reasonable personalized drug treatment plan, which can effectively reduce the mortality and morbidity of patients. Therefore, therapeutic regimens based on biomarkers have great potential and application in the treatment of Colon cancer (Pritzker, 2020). In recent times, Gan Boyi’s group and John Chen’s group have proposed disulfidptosis: cells with high SLC7A11 expression require regular NADPH supply, which is derived from oxidative glucose catabolism. If the glucose supply is cut off, NADPH will be reduced, leading to the accumulation of toxic disulfidptosiss and, eventually, cell death (Liu X. et al., 2023). The glucose deprivation triggers disulfidptosis, forming disulfidptosis bonds mainly in the actin cytoskeleton, particularly in cells with high SLC7A11 expression. Rapid NADPH depletion follows, a pivotal factor initiating disulfidptosis. The glucose deprivation also quickly alters the actin cytoskeleton, reducing protein migration, causing cell shrinkage, F-actin contraction, and membrane separation. Additionally, inhibiting glucose uptake with GLUT inhibitors replicates glucose starvation’s effects, promoting excessive disulfidptosis bond formation in the actin cytoskeleton and F-actin collapse, highlighting glucose metabolism’s role in disulfidptosis (Meng et al., 2023). With this discovery of a novel cell death mode caused by disulfidptosis accumulation, the medical community is expected to design new therapies targeting cancer cells, which have great potential for application in tumor therapy. LncRNAs play a critical role in regulating Colon cancer (CRC) by inhibiting tumor proliferation, metastasis, and controlling the growth, migration, and invasion of CRC cells both *in vivo* and *in vitro* (Li et al., 2018). However, the potential prognostic significance of DRLs in Colon cancer has not been investigated. Therefore, by studying Colon cancers carrying DRLs, a better understanding of the pathogenic mechanisms can be obtained, leading to earlier detection of lesions, risk stratification, and further improvement of patient survival. Our study successfully developed and validated a prognostic model for DRLs in Colon cancer. The feasibility of this prognostic model was confirmed through rigorous evaluation. We firmly believe that this multi-biomarker prognostic model, which is based on DRLs, holds great promise as an enhanced predictive tool in the medical field. Its potential to provide more accurate and reliable prognostic information is expected to actively contribute to improved treatment strategies for Colon cancer.

Through comprehensive analysis of transcriptomic data, clinical data and mutation data of COAD tumor samples in the TCGA database, we successfully identified four DRLs and their significance in assessing the prognosis and immune status of COAD patients. Through one-way Cox and machine learning analyses, we identified AC083900.1, AP003555.1, SNHG7, and ZEB1-AS1 as independent prognostic variables for COAD (Chi et al., 2023). By categorizing patients into low-risk and high-risk groups based on these DRLs, we achieved accurate categorization and demonstrated that their predictive performance was superior to traditional clinical indicators. Further analyses showed significant differences between the low and high-risk groups in terms of immune relevance, mutation profile, and drug sensitivity. These findings provide valuable insights for clinicians to make more informed decisions and improve overall survival outcomes for COAD patients. In addition, our validation using the disulfide model suggests that the four DRLs may be involved in the bisulfite death process, which further underscores their relevance in personalizing therapeutic strategies and improving the prognosis of COAD patients.

In recent years, an increasing number of long non-coding RNAs (lncRNAs) have been implicated in cancer development and progression. These lncRNAs play crucial roles in tumor cell proliferation, differentiation, and drug resistance, thereby influencing cancer development, progression, and therapeutic outcomes. As potential biomarkers for tumor prediction, they have attracted considerable attention (Han et al., 2019). In our study, we developed a prognostic model comprising four DRLs (AC083900.1, AP003555.1, SNHG7, and ZEB1-AS1) closely associated with the overall survival (OS) of COAD patients. Notably, ZEB1-AS1 expression was significantly upregulated in Colon cancer and correlated with the prognosis of Colon adenocarcinoma patients (Wei et al., 2022). Aberrant expression of ZEB1-AS1 is a critical driver of cancer cell invasion and metastasis. Through the miR-101/ZEB1 axis, ZEB1-AS1 promotes the proliferation and invasion of Colon cancer cells (Wu et al., 2018). Compared to normal tissues, ZEB1-AS1 expression was significantly elevated in Colon cancer tissues, while miR-101 levels were lower in Colon cancer tissues, exhibiting a negative correlation with ZEB1-AS1 and ZEB1 expression levels (Xiong et al., 2018). Consequently, targeting ZEB1-AS1 may impact tumor progression and metastasis, rendering it a promising biomarker. Furthermore, ZEB1-AS1 expression is correlated with tumor depth, degree of differentiation, lymph node and distant metastasis, clinical progression, and shortened survival, underscoring its pivotal role in tumorigenesis and development (Wu et al., 2018). SNHG7 gene expression has been found to be upregulated in various cancer types (e.g., bladder cancer, cervical cancer, Colon cancer) and is implicated as an oncogene involved in regulating cancer cell functions such as proliferation, apoptosis, invasion, and metastasis, thereby contributing to cancer cell survival and development. Additionally, SNHG7 can function as a competing endogenous RNA (ceRNA) to promote cancer development. Hence, SNHG7 is regarded as a significant cancer-associated biomarker (Zhang Y. et al., 2022). Recent studies have identified AP003555.1 as a prognostic marker for predicting the prognosis of Colon cancer (CRC) patients (Liu S. et al., 2023). Likewise, AC083900.1 has also been associated with CRC. Collectively, these findings enhance our understanding of COAD patients’ status and provide valuable insights and recommendations for their treatment.

To effectively demonstrate the signature function of DRLs, we performed an enrichment analysis of their pathways. As a result, we found that the signaling of DRLs is associated with many molecular functions, such as protein isoform activity and pigment structure composition, as well as pathways including systemic lupus erythematosus in cancer, alcohol toxicity, and the formation of neutrophil extracellular traps (NETs). Among these, loose extracellular chromatin fibers comprise the extracellular trap (Fang et al., 2022) formed by activated neutrophils decorated with excessive granules and cytoplasmic proteins. Recent studies have shown that elevated levels of neutrophil counts and NETs in the peripheral circulation are hallmarks of cancer. NETs can lead to a hypercoagulable state, accumulate in the peripheral vasculature and impair organ function, promote cancer development and metastasis, isolate circulating tumor cells, and even stimulate dormant cancer cells (Chu et al., 2021). Recent studies have found that elevated levels of neutrophil counts and NETs may be predictive of the presence of cancer. NETs are capable of promoting DTC proliferation and metastasis through the remodeling of the extracellular matrix (ECM). This remodeling process involves the cleavage of laminin, resulting in the formation of a new epitope that can be sensed by integral proteins on dormant cancer cells. Consequently, dormant cancer cells are activated, re-enter the cell cycle, and initiate invasive metastatic growth (Yang and Liu, 2021). This remodeling process involves the cleavage of laminin, resulting in the formation of a new epitope that can be sensed by integral proteins on dormant cancer cells. Consequently, dormant cancer cells are activated, re-enter the cell cycle, and initiate invasive metastatic growth.

The tumor microenvironment (TME) encompasses the contextual milieu surrounding tumor cells, comprising diverse constituents including blood vessels, immune cells, fibroblasts, and stroma. These elements engage in dynamic interactions, wherein the equilibrium between the immune system and the tumor intricately modulates this dynamic interplay (Bruschini et al., 2021; Wu et al., 2023). Comprehending immune cell infiltration within the TME holds pivotal significance in elucidating tumor immune evasion mechanisms and facilitating insights into tailored therapeutic approaches. Enhanced understanding of immune infiltration in the tumor microenvironment can optimize treatment protocols, ultimately yielding improved clinical outcomes (Wu et al., 2023; Yang et al., 2023). Understanding the immune cell infiltration within the TME is crucial for comprehending the immune evasion mechanisms of tumors and providing insights into personalized treatment strategies. By gaining in-depth knowledge of immune infiltration in the tumor microenvironment, treatment regimens can be optimized, leading to improved clinical outcomes (Chen et al., 2022; Yu et al., 2022). It has been shown that the activation of tumor-infiltrating CD8T cells is significantly increased after resveratrol treatment. Resveratrol activates CD8T cells against tumors by stimulating IL-18 derived from macrophages (Zhang W. et al., 2022). Therefore, the use of resveratrol to activate CD8^+^ T cells in high-risk populations holds substantial therapeutic potential. CD8^+^ T lymphocytes have a positive impact on various types of cancer, and their study and development in cancer therapy carry significant implications (Li L. Q. et al., 2020).

High mutational load is characterized by an elevated number of genetic alterations in tumor cells relative to their normal counterparts. This condition is intricately linked to the amplification of tumor antigenic or immunogenic neoantigens. Consequently, it promotes the recruitment of tumor-infiltrating lymphocytes, including CD8^+^ T cells, which exhibit a notable correlation with high mutational load (Kaumaya, 2020). Cancer cells often have new mutated genes that are better antigens. Therefore the discovery of these widely mutated neoantigens is significant for targeted therapy in COAD. Colon cancer cells are usually characterized by abnormalities in chromosome number and quality. The wild-type APC acts as a bridge between microtubules and chromosomes, forming a complex with the mitotic checkpoint proteins Bub1 and Bub3, which help promote proper growth of spindle formation and maintain haploidy. If the APC gene is mutated, truncated proteins may lose their ability to bind to Bub1 and fail to properly maintain microtubule attachment to the kinetochore, leading to defects in chromosome segregation, which then leads to poor prognosis in Colon cancer patients (Narayan and Roy, 2003). TP53 is an important tumor suppressor gene. Statistically, TP53 gene mutations are frequently observed in a variety of cancers, including Colon, breast, and bile duct cancers (Takano et al., 2016). TP53 mutations predominantly occur within exons five to eight, encompassing the core structural domain responsible for the folding and stability of the p53 protein. Notably, residues 130–286 within this region play a pivotal role in maintaining p53’s DNA-binding capacity, and mutations in this domain lead to the loss of p53’s functionality (Al-Kuraya, 2009). The TTN gene encodes myosin, a crucial protein involved in vertebrate rhabdomyolysis, contributing to its structural and functional integrity (Zhang Y. et al., 2022). While the involvement of the TTN gene in cancer biology remains contentious, emerging evidence suggests its immunological relevance. Mutated TTN peptides have demonstrated the ability to elicit CD8^+^ T cell responses, albeit at a lower frequency compared to other tested antigens (Rus Bakarurraini et al., 2020). The identification of highly mutated genes in COAD presents novel avenues for targeted therapies, holding promise for improved clinical outcomes.

Cell culture and qPCR assay played a key role in this study. We selected Caco-2 and SW480 colon cancer cells and NCM460 normal breast cells. Culture conditions included DMEM medium supplemented with 10% fetal bovine serum, 100 U/L penicillin, and 100 mg/L streptomycin to ensure cell growth and maintenance. RNA extraction was performed using the RNA Eazy Fast Tissue/Cell Kit, followed by cDNA synthesis using the FastKing RT Kit. We performed a real-time PCR procedure consisting of an initial pre-denaturation step followed by 40 cycles including denaturation, annealing and extension steps. Transient transfection was performed in order to study gene regulation. Cell viability was determined by CCK-8 assay, which reflects the metabolic activity and viability of the cells by OD450 value. This assay was used for comparison between different experimental conditions and treatment groups to provide quantitative data on relative cell viability. Finally, the migratory and invasive capacity of the cells was assessed by Transwell assay, which provided important information about cell behaviour. These experimental methods contribute to a deeper understanding of the molecular mechanisms and cellular behaviours of colon cancer and provide a solid experimental basis for the findings.

While our prognostic model has demonstrated significant utility in predicting Colon cancer patient outcomes and aiding treatment decision-making, it is important to acknowledge the limitations of our study. Firstly, the reliance on data from public databases introduces the potential for disparities between predicted outcomes and real-world conditions. Therefore, it is imperative to obtain prospective clinical information and sequencing data following immunotherapy to validate the clinical efficacy of our model. Secondly, inter-individual variations among COAD patients may influence the characteristics of the four identified DRLs. Although we attempted to validate our findings using external datasets such as GEO and ICGC, the inherent biases and limitations associated with microarray data may have hindered the acquisition of accurate LncRNA information. Addressing these limitations and enhancing the robustness of our model will require the development of novel strategies and further research endeavors.

## 5 Conclusion

In conclusion, our study successfully identified specific long non-coding RNAs (lncRNAs) that are closely associated with death by disulfide, revealing new prognostic biomarkers and potential therapeutic targets for colon cancer (COAD) patients. In addition, we utilized these lncRNAs to develop a predictive model that provides accurate prognostic assessment for COAD patients and valuable insights into their immune status. This model is expected to optimize personalized systemic therapy for COAD patients.

## Data Availability

The original contributions presented in the study are included in the article/Supplementary Material, further inquiries can be directed to the corresponding authors.
