# Supplementary figures and images for: Unraveling the role of disulfidptosis-related LncRNAs in colon cancer: a prognostic indicator for immunotherapy response, chemotherapy sensitivity, and insights into cell death mechanisms
Source: Front Mol Biosci. 2023 Oct 17;10:1254232. doi: 10.3389/fmolb.2023.1254232 (PMC10617599; doi:10.3389/fmolb.2023.1254232)

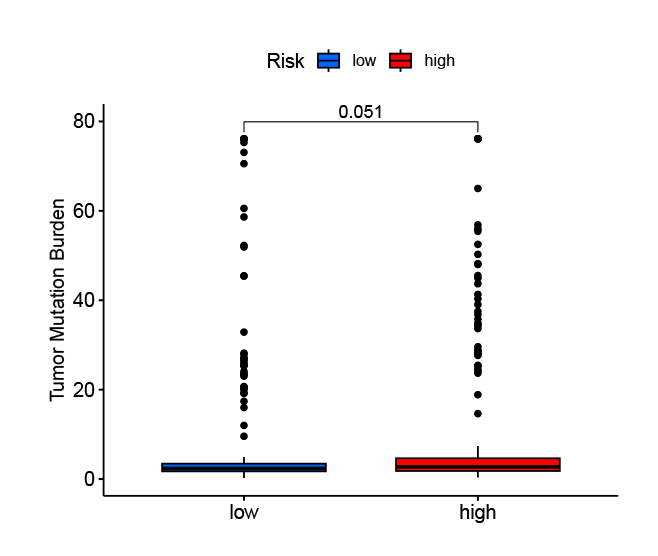

Supplement: Supplementary file 1 [file Image2.TIF]

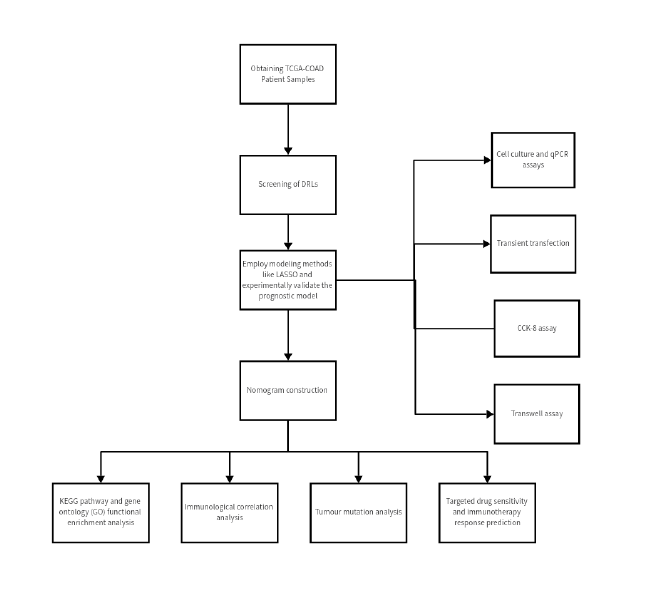

Supplement: Supplementary file 2 [file Image1.TIF]
